# Plant Extracts Target Biofilms to Alleviate Nutritional and Metabolic Disorders in Animals

**DOI:** 10.3390/vetsci13040337

**Published:** 2026-03-31

**Authors:** Yang Wang, Shenao Song, Shuji Gao, Xiaoling Zhang, Yuxin Wang, Baobao Liu

**Affiliations:** 1 College of Animal Science and Technology, Henan University of Science and Technology, Luoyang 471000, China; 2 Henan Provincial Engineering Research Center for Detection and Prevention and Control of Emerging Infectious Diseases in Livestock and Poultry, Luoyang 471003, China

**Keywords:** biofilm, virulence, plant extracts

## Abstract

Bacterial biofilms represent a major driver of persistent infections in animals, productivity losses, and increased antibiotic use. The chronic inflammation and metabolic dysregulation induced by biofilms constitute major predisposing factors for nutritional and metabolic disorders in veterinary settings. Conventional antibiotic therapy is often limited by reduced efficacy and the emergence of resistance in biofilm eradication. Moreover, drug residues and associated metabolic toxicity pose additional risks to animal health and food safety. Plant extracts, characterized by multi-target modes of action, favourable biocompatibility, and metabolic regulatory potential, represent a sustainable strategy for addressing this challenge. This narrative review systematically examines the mechanisms by which plant-derived compounds modulate the formation and dispersal of pathogenic biofilms via direct and indirect pathways.

## Simple Summary

Bacterial biofilms are communities of microorganisms that attach to surfaces and form a protective barrier, making infections difficult to treat. In animals, biofilms cause persistent diseases, reduce growth and productivity, and increase the need for antibiotics. Traditional treatments often fail due to drug resistance and the strong structure of biofilms. Plant extracts, which contain natural bioactive compounds, offer a promising alternative. They can disrupt biofilms by directly damaging bacterial cells or by interfering with bacterial communication and regulatory systems. This review summarizes how plant derived compounds work against biofilms and highlights their potential to improve animal health and reduce reliance on antibiotics.

## 1. Introduction

Pathogenic biofilms are complex three-dimensional structures formed by microbial communities adhering to biotic or abiotic surfaces and encased within a self-produced extracellular polymeric matrix. They represent a major contributor to chronic infections [1]. Biofilms are widely present in natural environments, medical devices, food processing equipment, and human tissue surfaces, closely associated with various chronic infections and persistent contamination. More than 80% of environmental bacteria are estimated to exist in biofilms and exhibit antibiotic tolerance that is 10–1000 fold higher than that of planktonic cells [2]. Biofilms can evade host immune clearance, resulting in persistent clinical infections associated with elevated mortality. In oral infections, device-associated infections, and foodborne diseases [3]. In conditions such as mastitis in dairy cows, respiratory infections in swine and poultry, oral infections in companion animals, and device-associated infections in veterinary practice, biofilm formation constitutes a major determinant of disease persistence, recurrence, and treatment failure [4]. The relationship between biofilm formation and metabolic dysfunction in animals represents an emerging area of investigation with profound clinical implications. Chronic biofilm infections establish a persistent inflammatory state characterized by sustained activation of innate immune responses, including neutrophil infiltration, macrophage activation, and continuous production of pro-inflammatory cytokines such as tumor necrosis factor-alpha (TNF-α), interleukin-1 beta (IL-1β), and interleukin-6 (IL-6) [5]. This chronic inflammatory environment exerts substantial metabolic costs on the host. Prolonged inflammation drives a state of negative energy balance, as immune activation requires considerable nutrient and energy resources while simultaneously suppressing appetite and altering nutrient partitioning [6,7]. In production animals, this metabolic burden manifests as reduced weight gain, decreased feed efficiency, and impaired reproductive performance. In dairy cattle, for instance, biofilm-associated intramammary infections not only reduce milk yield but also alter milk composition and increase somatic cell counts, leading to substantial economic losses [8]. Quantitatively, mastitic cows have been shown to have a calving to first interval 6.1 days longer, and produce 549.6 kg less milk and 20.4 kg less fat per 305 day lactation, compared with non-mastitic cows [9]. Furthermore, systemic inflammatory mediators disrupt endocrine signaling pathways involved in metabolic regulation, including insulin signaling, growth hormone axis function, and thyroid hormone metabolism, thereby contributing to the development of metabolic disorders such as ketosis, fatty liver syndrome, and insulin resistance [10,11].

Current strategies for biofilm prevention and control include physical removal, chemical disinfection, and biological intervention. However, antibiotic therapy is often constrained by the structural barriers and metabolic heterogeneity of biofilms (i.e., spatial and temporal variation in metabolic activity among cells within the biofilms), and prolonged use promotes resistance development. Chemical disinfectants, while partially effective in biofilm removal, pose issues such as toxic residues, environmental pollution, and equipment corrosion [12,13]. Physical methods, such as ultrasound and ultraviolet irradiation, are often used as adjunctive treatments but are insufficient for thorough eradication [14,15,16], and their application is largely impractical for most in vivo veterinary uses. In this context, plant extracts have emerged as a new research hotspot in anti-biofilm studies due to their wide availability, structural diversity, multi-target mechanisms, and environmental friendliness. Plants contain diverse bioactive compounds including flavonoids, polyphenols, terpenoids, and alkaloids that exert antimicrobial effects and modulate biofilm formation and maturation through mechanisms such as quorum sensing interference, inhibition of extracellular polysaccharide synthesis, and structural disruption [17]. Recent studies highlight the substantial role of plant-derived compounds in biofilm control [18]. For instance, flavonoids such as quercetin and apigenin reduce extracellular polysaccharide synthesis by inhibiting glycosyltransferase in *Streptococcus mutans* (*S. mutans*) [19]. Phenolics including curcumin and cinnamaldehyde disrupt quorum sensing and inhibit biofilm-associated gene expression [20], and terpenoids such as farnesol alter surface properties and impair adhesion [21]. These findings deepen mechanistic understanding of plant-mediated biofilm attenuation and provide a basis for developing plant-derived anti-biofilm therapeutics.

This review systematically summarizes the mechanisms and recent advances in plant-extract-mediated attenuation of pathogenic biofilm virulence, with a focus on their application in veterinary settings. It highlights the roles of plant-derived compounds in controlling biofilms associated with common animal pathogens and evaluates their potential to mitigate biofilm-related infections in livestock and poultry production. Amid global public health challenges such as antimicrobial resistance and the growing demand for sustainable animal husbandry, leveraging plant resources for anti-biofilm applications offers a promising strategy to reduce reliance on conventional antibiotics, improve animal health and productivity, and advance the integration of traditional medicinal plants into modern veterinary practice.

## 2. Review Methodology

### 2.1. Study Design

The variety of study designs (laboratory experiments and mechanistic in vitro studies) and the diversity of analytical methods allowed this work to be presented as a narrative review supported by a systematic literature search (but not a formal systematic review or meta-analysis).

### 2.2. Databases and Search Strategy

To this end, PubMed/MEDLINE and Web of Science Core Collection were searched, and Google Scholar was additionally screened to identify grey literature and recently published articles not yet indexed in bibliographic databases, with database searches conducted until 20 February 2026, and reference lists of key reviews and highly relevant primary studies manually checked to identify further eligible articles.

### 2.3. Search Terms

Search terms comprised a combination of controlled vocabulary (where applicable) and free-text terms related to bacterial biofilms, plant extracts, and agricultural/veterinary applications, with example query blocks including (biofilm OR bacterial biofilm OR biofilm-associated infection) AND (plant extract OR phytochemical OR herbal medicine OR peony) AND (veterinary OR livestock OR poultry OR dairy OR animal husbandry OR production animal).

### 2.4. Inclusion and Exclusion Criteria

Articles were deemed eligible if they met the following criteria: (i) they addressed biofilm-associated infections caused by pathogenic bacteria in livestock or poultry; or (ii) they reported on the effects of plant extracts or phytochemicals against bacterial biofilms using in vitro models with clear documentation of the experimental conditions and outcomes. Studies that were not related to animal-associated pathogens, or that lacked sufficient information on the identity, preparation, or concentration of the plant extract, were excluded.

### 2.5. Selection and Synthesis of Studies

The selection of studies was performed by screening titles and abstracts, followed by full-text assessment of the remaining articles, and synthesis of evidence was conducted qualitatively, with studies categorized according to the proposed mode of action of the plant extracts (direct vs. indirect) and the biofilm model used. Where two studies addressed the same research question, preference was given to those with a more clearly defined experimental design, more rigorous characterization of the extract’s activity, and greater relevance to livestock production systems, while seminal studies and key guidance documents were retained when they influenced the conceptual framework or interpretation. The graphics of the article are drawn using Adobe Illustrator 2022.

## 3. Biofilms

Biofilms are surface-associated microbial aggregates embedded within a self-produced extracellular polymeric matrix composed of polysaccharides, proteins, and extracellular DNA [3]. Biofilm formation represents a fundamental microbial survival strategy. Biofilm-associated traits protect microorganisms from environmental stress, enhance tolerance to antimicrobial agents, and promote persistence and virulence [22]. Moreover, when colonizing biodegradable surfaces, biofilms can facilitate nutrient acquisition through gradual substrate degradation.

### 3.1. Biofilm Formation

**Figure 1:**
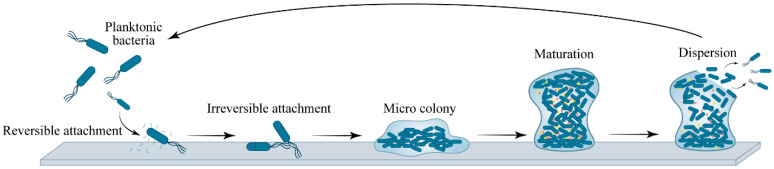
Biofilm formation process.

Biofilm formation is a dynamic and highly ordered process that begins with the exploration and surface contact of planktonic cells. This developmental cascade follows a generally conserved sequence across bacterial species, though specific molecular details vary depending on the organism and environmental context. Understanding these stages provides an essential foundation for appreciating how plant extracts can intervene at multiple points to mitigate biofilm-associated metabolic disorders. The biofilm life cycle begins when planktonic bacteria encounter a suitable surface—which may be biological tissue, such as epithelial linings of the respiratory or gastrointestinal tract, or abiotic surfaces, including medical devices, feeding equipment, or water systems in animal production facilities. Bacteria approach surfaces through multiple mechanisms, with motility structures playing particularly important roles. Flagella enable bacteria to overcome repulsive forces between the cell and surface and facilitate initial contact [23]. Upon approaching a surface, bacteria establish transient interactions mediated by weak physicochemical forces, including van der Waals interactions, electrostatic forces, and hydrophobic effects [24,25]. Bacteria that remain in contact with a surface for sufficient duration transition to irreversible adhesion, a critical commitment step in biofilm development. During this phase, cells undergo substantial physiological changes, including transcriptional reprogramming that shifts gene expression patterns from those characteristics of planktonic growth to those supporting surface-associated lifestyles [26]. Irreversible adhesion is stabilized by production of specific adhesins, surface proteins and polysaccharides that mediate strong binding to surface components. Concurrently, bacteria begin secreting small quantities of extracellular polymeric substances that anchor cells more firmly to the surface and to each other (Figure 1). Following stable surface adhesion, bacteria begin to proliferate, forming small clusters termed microcolonies. As cell numbers increase, they initiate large-scale synthesis and secretion of extracellular polymeric substances (EPS), which constitute the structural scaffold of mature biofilms. EPS is a complex mixture of biopolymers, primarily composed of polysaccharides, proteins, and extracellular DNA (eDNA) [27]. The polysaccharide components maintain structural cohesion and create diffusion barriers protecting embedded cells. Specific polysaccharides vary among species, for example, *Pseudomonas aeruginosa* produces alginate, Pel, and Psl polysaccharides [28], while *Staphylococcus aureus* produces polysaccharide intercellular adhesin and *Streptococcus mutans* synthesizes water-insoluble glucans from dietary sucrose [29,30].

As microcolonies expand and coalesce, they develop into mature biofilms characterized by complex three-dimensional architectures. These structures often contain mushroom- or tower-like formations separated by fluid-filled channels that facilitate transport of nutrients to deeper regions and removal of metabolic waste products. The development of this architecture is actively regulated through intercellular signaling systems, particularly quorum sensing. The mature biofilm represents a functional microecosystem in which distinct subpopulations of bacteria assume different roles. Cells at the biofilm periphery exhibit relatively high metabolic activity, while cells deep within the biofilm experience oxygen limitation and nutrient deprivation, entering slow-growing or dormant states characterized by reduced susceptibility to antimicrobial agents [31]. Ultimately, biofilms enter a dispersal phase triggered by nutrient depletion, metabolic waste accumulation, or signaling molecules such as low concentrations of nitric oxide or D-amino acids [32,33]. During this phase, bacteria detach actively or passively through matrix degradation or altered motility, revert to a planktonic state, and colonize new surfaces, thereby initiating another cycle of biofilm development (Figure 1).

### 3.2. Regulatory Mechanisms of Biofilm Formation

Biofilm formation is not simply a passive accumulation of bacteria but an active developmental process governed by a complex molecular network. Central to this regulation is the ability to sense environmental cues including nutrient availability, stress, surface contact, population density, and coordinate gene expression during the transition from planktonic growth to community organization. This network operates across multiple regulatory layers, encompassing second messenger systems represented by c-di-GMP, cell communication systems like quorum sensing (QS), and post-transcriptional regulators such as small RNAs (sRNAs). These components interact synergistically to orchestrate biofilm development and dispersal [34].

c-di-GMP acts as a central regulator of the transition between motile and sessile lifestyles. Intracellular concentrations are controlled by diguanylate cyclases (DGCs, containing GGDEF domains) and phosphodiesterases (PDEs, containing EAL or HD-GYP domains). Elevated c-di-GMP levels generally promote biofilm formation by inhibiting transcriptional regulators such as FleQ, repressing flagellar gene expression and reducing motility, thereby facilitating surface colonization by limiting active detachment from the surface [35,36,37]. c-di-GMP also activates proteins involved in extracellular polysaccharide synthesis. In *Pseudomonas aeruginosa* (*P. aeruginosa*), elevated levels bind PelD or convert FleQ into an activator to promote pel operon expression [38]. Simultaneously, c-di-GMP binding to the PilZ domain of the Alg44 protein is essential for the overproduction of alginate in mucoid strains [39]. Moreover, c-di-GMP regulates collective behaviors such as swarming by regulating enzymes including SadC and BifA, refining early colonization [40]. Conversely, dispersal cues such as nutrient limitation or nitric oxide upregulate PDEs (e.g., DipA and RbdA), reducing intracellular c-di-GMP [41]. Reduced c-di-GMP restores motility and can activate matrix-degrading enzymes via regulators such as BdlA, facilitating detachment and planktonic reversion [42].

Quorum sensing (QS) system functions as a “census-taking” mechanism for bacterial populations, enabling them to sense population density and synchronize group behaviors. When autoinducers such as AHLs or AIPs accumulate to threshold levels, receptor binding initiates regulatory cascades coordinating expression of biofilm-associated genes [43]. In *P. aeruginosa*, hierarchical Las, Rhl and PQS networks regulate rhamnolipid production for structural remodeling and indirectly modulate c-di-GMP metabolism [44]. For example, QS-regulated *tpbA* expression influences c-di-GMP levels and Pel synthesis. In *Staphylococcus aureus*, Agr QS downregulates the expression of surface adhesins (e.g., fibronectin-binding proteins) while upregulating toxin production, such as phenol-soluble modulins (PSMs, a family of surfactant-like peptides originally named for their isolation method), thereby promoting biofilm decomposition, facilitating late-stage dispersal [45]. Additionally, QS influences the composition of the biofilm extracellular matrix. In *P. aeruginosa*, PQS induces quinolone-mediated cell lysis releasing eDNA that stabilizes matrices and chelates cations, enhancing antibiotic tolerance [46].

Beyond second messenger systems and QS, other regulatory factors also play critical roles. Two-component systems (TCS) act as environmental sensors, converting external stimuli into intracellular signals. Small RNAs mediate rapid post-transcriptional responses, including modulation of enzymes controlling c-di-GMP metabolism through regulators such as RNA-binding proteins like CsrA, contributing to the fine-tuning of biofilm formation [47,48]. The second messenger (p)ppGpp participates in biofilm dispersal decisions during nutrient stress by influencing c-di-GMP levels or directly regulating gene expression [49,50]. This integrated regulatory architecture enables adaptation to environmental stress, resource acquisition, and immune or antimicrobial evasion via multicellular organization. It also provides numerous therapeutic targets, including disruption of c-di-GMP signalling or QS quenching strategies.

## 4. Strategies of Plant Extracts Against Biofilms

The development of effective strategies for combating biofilm-associated infections requires comprehensive understanding of how plant extracts interfere with the complex biology of these microbial communities. Plant extracts have attracted increasing attention owing to their sustainability, low toxicity, and ability to target biofilms through synergistic, multi-target mechanisms that distinguish them from conventional antimicrobial agents [51,52]. Unlike antibiotics that typically inhibit single essential bacterial functions, plant-derived compounds simultaneously affect multiple cellular targets, reducing the likelihood of resistance development and offering potential for efficacy against established biofilms that resist conventional treatment [53,54]. Based on their primary modes of action, the anti-biofilm activities of plant extracts can be broadly categorized into two principal classes: direct action and indirect action. Direct action involves bioactive compounds physically or chemically interacting with bacterial cells or biofilm matrix components, disrupting structural integrity or function through mechanisms such as membrane damage, enzyme inhibition, or matrix degradation [55]. These effects occur independently of bacterial regulatory networks and typically manifest rapidly upon exposure. Indirect action, by contrast, involves interference with bacterial signaling and regulatory systems including quorum sensing, second messenger cascades, and two component signal transduction, thereby modulating gene expression and community behavior without necessarily causing immediate cell death [56]. This distinction, while useful for conceptual clarity, should be recognized as somewhat artificial, as many plant extracts exhibit both direct and indirect effects that may synergize to produce enhanced anti-biofilm activity [57,58]. The following sections examine each category in detail, highlighting representative compounds and their mechanisms of action against pathogenic bacterial biofilms.

### 4.1. Direct Action

**Figure 2:**
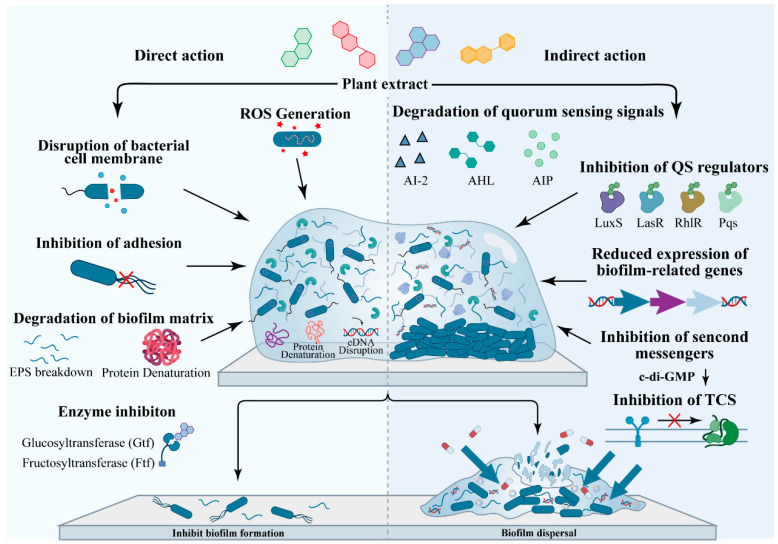
Mechanism of plant extracts on biofilm of pathogenic bacteria.

Direct action represents the most intuitive and rapid approach for plant extracts in combating biofilms. These mechanisms involve bioactive constituents disrupting bacterial cellular structures, destabilizing the biofilm matrix, or inhibiting essential enzymatic activities, thereby reducing bacterial adhesion, aggregation, and persistence [59,60,61]. Such activity operates independently of complex bacterial regulatory networks and is characterized by rapid onset and defined molecular targeting. Many bioactive constituents in plant extracts directly damage bacterial cell envelopes, resulting in leakage of intracellular contents, cell death, or functional impairment, thereby arresting biofilm development at early stages. For instance, polyphenols (e.g., catechins, gallic acid) and related phenolics (e.g., curcumin) insert into membrane phospholipid bilayers, disrupting fluidity, increasing permeability, and inducing ionic imbalance and metabolic dysfunction [62,63]. Epigallocatechin gallate (EGCG) generates reactive oxygen species (ROS), inducing oxidative stress that compromises the integrity of *Staphylococcus aureus* membranes and leads to marked depletion of intracellular ATP and loss of viability (Figure 2) [64]. Additionally, alkaloids (e.g., berberine) and terpenoids (e.g., menthol, *Eucalyptus camaldulensis* ethanolic leaf extract) exhibit pronounced surface activity that solubilizes membrane lipids and promotes structural disruption (Figure 2) [65,66,67]. Such cytotoxicity affects both planktonic cells and bacteria embedded within biofilm matrices. For instance, terpenes from tea tree oil penetrate the extracellular polysaccharide barrier of *P. aeruginosa* biofilm, targeting deeply embedded cells and demonstrating strong eradication potential [68]. Bacterial adhesion represents the initial step of biofilm formation and depends on motility structures, including flagella and pili, as well as surface adhesins. Plant extracts can directly inhibit these structures and associated proteins, thereby preventing bacterial adhesion. Flavonoids (e.g., kaempferol) and alkaloids (e.g., piperine, reserpine) interfere with the assembly or energy provision of flagellar motor proteins, reducing chemotactic motility and swimming capacity [69]. For instance, carvacrol significantly reduces flagellar protein expression and swimming motility in *Escherichia coli*, *Salmonella*, and *Campylobacter* species [70,71,72]. Flavonoid derivatives, including chalcone and morin, form covalent adducts with cysteine residues at the active site of SrtA enzyme, irreversibly inhibiting its activity. This diminishes the presentation of key adhesins, such as AgI/II and SpaP, on the cell wall of *S. mutans*, markedly diminishing bacterial adherence to host surfaces (e.g., tooth enamel) [73]. *Torilis japonica* ethanol extracts inhibit the expression of adhesion genes *sarA*, *icaA*, and *hla* in *Staphylococcus aureus* KCTC 1927, inhibiting biofilm formation [74].

Extracellular polysaccharides (EPS), which constitute a major structural component of biofilms, are primarily synthesized by enzymes including glucosyltransferases (Gtfs) and fructosyltransferases (Ftfs). Several flavonoids (e.g., quercetin, apigenin) and phenolic acids (e.g., salicylic acid, chlorogenic acid) act as competitive or non-competitive inhibitors of these enzymes [75,76,77]. For instance, quercetin binds specifically to the active sites of GtfB and GtfD in *S. mutans*, inhibiting the synthesis of water-insoluble glucans (Figure 2) [78]. Chlorogenic acid targets AlgD, an enzyme involved in alginate synthesis, thereby reducing EPS production and inhibiting biofilm formation in *P. aeruginosa* [79]. Certain plant extracts either contain or stimulate the production of enzymatically active compounds capable of directly degrading polysaccharides, proteins, or eDNA within the biofilm matrix. For instance, tannin-rich plant extracts (e.g., pomegranate peel extract) generate hydrolytic products that bind to matrix proteins, inducing denaturation and inactivation [80,81]. Specific plant saponins exhibit surfactant-like properties, lowering matrix surface tension and facilitating dissociation. Examples include theasaponin E1 (TE1), theasaponin E2 (TE2), and assamsaponin A (ASA), which modulate bacterial cell surface hydrophobicity, adhesion ability, and phospholipase activity, ultimately inhibiting *Candida albicans* biofilm formation [82]. Biofilm matrix stability depends on multiple intermolecular forces, including hydrogen bonds, hydrophobic interactions, and ionic interactions. Certain plant polyphenols and quinones (e.g., emodin, hypericin) integrate into the matrix network, competitively binding to critical sites and weakening these forces. Consequently, the matrix becomes less dense, with increased porosity, enhancing the penetration of antibiotics and other antimicrobial agents [83,84].

Overview of the therapeutic effects of known plant extracts against pathogenic bacterial biofilms, including inhibition of biofilm formation and disruption of mature biofilms. The mechanisms are summarized according to direct and indirect actions. Direct actions include disruption of cell wall integrity, inhibition of adhesion, degradation of the biofilm matrix, and induction of reactive oxygen species (ROS). Indirect actions include inhibition of quorum sensing (QS) systems, degradation of QS signaling molecules, and interference with second messenger systems.

### 4.2. Indirect Action

Unlike direct action, indirect action does not cause bacterial death or directly disrupt the extracellular matrix. Instead, they impede biofilm formation or facilitate the disassembly of mature biofilms by modulating QS, regulating intracellular second messenger concentrations, and altering stress responses and metabolic states, acting primarily at the levels of signal transduction and gene regulation. Such strategies are less prone to induce strong resistance and may exert distinctive, multi-target effects on persistent bacterial populations embedded within mature biofilm, acting through mechanisms such as quorum quenching, disruption of matrix integrity, and metabolic interference, rather than through direct bactericidal activity alone. QS represents a fundamental bacterial communication mechanism by which populations coordinate collective behaviors, including biofilm formation, in response to population density. Plant extracts constitute a rich source of quorum sensing inhibitors (QSIs) and can disrupt QS through multiple mechanisms. The AI-2 QS system is a widely conserved interspecies signaling mechanism. Studies have demonstrated that compounds such as paeoniflorin, derived from peony, and methyl anthranilate, extracted from grape and strawberry, competitively bind to the LuxS protein, inhibiting its enzymatic activity and thereby reducing AI-2 signal molecule production. Consequently, this inhibits the transcription of AI-2 QS-dependent adhesion and virulence genes, ultimately inhibiting biofilm formation by *Streptococcus suis * (Table 1) [52,85]. Curcumin displays potent QSI activity across diverse bacterial species. Specifically, curcumin inhibits the AI-2 QS of *B. subtilis* by 77% (at 5 µg/mL) and the LasI/LasR QS system of *P. aeruginosa* by 21% (at 200 µg/mL) (Figure 2) [86]. Furthermore, Hayder et al. developed a curcumin-mediated antimicrobial photodynamic therapy. Combined treatment with curcumin and 10 J/cm^2^ light decreased EPS production in *P. aeruginosa* by 74%, markedly inhibiting biofilm formation (Table 1) [87]. Furanones, including brominated furanone C-30 extracted from algae, alongside select flavonoids, are classical antagonists of acyl-homoserine lactone (AHL) receptors. These compounds effectively inhibit the LasR/RhlR QS of *P. aeruginosa*, resulting in downregulation of virulence factors and adhesion genes, thus inhibiting biofilm formation [88]. Moreover, C-30 exhibits pronounced biofilm-inhibitory effects against *S. mutans* [89]. Chlorogenic acid disrupts the Las, PQS, and Rhl QS and modulates β-alanine and pyrimidine metabolism, collectively inhibiting *P. aeruginosa* biofilm formation [90]. Pomegranate peel extract and its principal constituent, punicalagin, target and bind LasR, antagonize QS, inhibit *P. aeruginosa* swarming, and ultimately inhibit biofilm formation [91]. Certain plant-derived components activate relevant enzyme activities, accelerating the degradation of c-di-GMP. For instance, citrus peel extract from Jeju Island (CPEJ) enhances phosphodiesterase activity, accelerates c-di-GMP degradation, and consequently inhibits biofilm formation [92]. Cranberry extract modulates c-di-GMP levels, reduce matrix production and secretion, and ultimately impedes the development and maturation of *Vibrio cholerae* biofilm [93]. Two-component systems also serve as critical hubs for bacterial environmental sensing and regulation of gene expression. 20S-ginsenoside Rg3 (Rg3), derived from red ginseng in traditional Chinese medicine, inhibits the SaeR/SaeS two-component system, thereby reducing bacterial adhesion and aggregation and ultimately inhibiting *Staphylococcus aureus* biofilm formation (Figure 2) (Table 1) [94].

**Table 1:** Plant extracts with demonstrated inhibitory or eradicating effects on pathogenic bacterial biofilm.

Plant Extracts	Bacteria	Mechanism	References
Apigenin	*Streptococcus mutans*	Inhibit glycosyltransferase activity and reduce EPS synthesis	[76]
Quercetin	*Streptococcus mutans*/*Staphylococcus epidermidis*	Specifically binding to the active sites of GtfB and Gtfd enzymes, inhibiting EPS synthesis	[78]
Curcumin	*Pseudomonas aeruginosa*/*Bacillus subtilis*	Block quorum sensing and inhibit the expression of biofilm related genes	[86,87]
Cinnamaldehyde	*Enterococcus faecalis*		[95]
Epigallocatechin	*Staphylococcus aureus*	Damage to cell membrane integrity	[64]
Kaempferol	*Staphylococcus aureus*	Inhibition of aggregation and adhesion	[69]
Piperine	*Streptococcus mutans*/*Staphylococcus aureus*		[96,97]
Chalcone	*Streptococcus mutans*	Inhibit the activity of srtA enzyme and reduce the display of adhesin	[73]
Chlorogenic acid	*Pseudomonas aeruginosa*	Interference with LAS, PQS, RHL quorum sensing system and metabolism	[79,90]
Salicylic acid	*Staphylococcus aureus*/*Escherichia coli*	Inhibit Agr QS	[77,98]
Paeoniflorin	*Streptococcus suis*	Interference of AI-2 QS, inhibition of adhesion gene expression and EPS synthesis	[52]
Methyl anthranilate	*Streptococcus suis*	Interference of AI-2 QS, inhibition of adhesion gene expression and EPS synthesis	[85]
Furanone C-30	*Pseudomonas aeruginosa*/*Streptococcus mutans*	Interfere with lasr/rhlr system and AI-2 QS, inhibit adhesion	[88,89]
20s Ginsenoside Rg3	*Staphylococcus aureus*	Inhibit the Saer/Saes two-component system and reduce bacterial adhesion and aggregation.	[94]
Caffeic acid	*Streptococcus mutans*	Destroy the cell membrane and reduce the metabolic activity of the cell	[99]
Catechin	*Streptococcus mutans*	Cell wall structure of ring breaking bacteria, inhibiting adhesion	[100]
Allicin	*Escherichia coli*	Inhibition of adhesion and swimming	[101]
Isokaurenic acid	*Streptococcus mutans*	Inhibition of growth and acid production	[102]
Curcumol	*Streptococcus mutans*	Inhibition of adhesion	[103]
Farnesol	*Streptococcus mutans*	Promote glycolysis	[104]

## 5. Outlook

Plant extracts, representing a rich repository of agents against pathogenic bacterial biofilms, are emerging as a strategic frontier for addressing biofilm-associated challenges owing to their diverse chemical composition, multi-target modes of action, and favorable biocompatibility. Recent studies have yielded significant insights into biofilm molecular targets, elucidated key signaling pathways, and demonstrated the in vitro efficacy of specific extracts, thereby laying the groundwork for future applications. Nevertheless, the translation of laboratory findings into clinical or industrial applications remains constrained by several bottlenecks. First, the mechanistic depth and synergistic networks underlying biofilm inhibition require further elucidation. Although most investigations examine single components targeting individual pathways, natural extracts function as complex ‘chemical cocktails’ in which synergistic interactions among multiple constituents and their interplay with the host microenvironment remain largely unexplored. Emerging evidence suggests that combinations of plant compounds can produce effects substantially greater than the sum of their individual activities, through mechanisms including enhanced membrane penetration, inhibition of bacterial efflux pumps, and simultaneous targeting of complementary pathways [105]. For example, the combination of flavonoids and terpenoids in essential oils often exhibits enhanced anti-biofilm activity compared to either class alone, likely reflecting their complementary actions on membrane integrity and intracellular targets [106]. Understanding these synergistic networks could enable the development of optimized extract combinations or formulations that maximize therapeutic efficacy while minimizing required doses and potential toxicity. Furthermore, the interplay between plant extracts and the host microenvironment represents a critical knowledge gap. Biofilm infections in vivo exist within complex host contexts that include immune cells, tissue factors, nutritional status, and commensal microbial communities, all of which can significantly influence both biofilm behavior and the activity of therapeutic agents [107]. Plant extracts may modulate host immune responses, tissue repair processes, and metabolic pathways in ways that synergize with their direct anti-biofilm effects, yet these interactions remain poorly characterized.

Second, both efficacy and specificity necessitate precise optimization. Numerous components display cytotoxicity at effective doses or possess a limited antimicrobial spectrum, thus urgently requiring structural modifications, nano-encapsulation, or alternative strategies to enhance targeting and therapeutic windows. Third, the capacity to eradicate mature and structurally complex biofilms remains inadequate. The dense extracellular matrix of mature biofilms and the persistence of embedded bacterial populations constitute a formidable barrier, necessitating the development of advanced strategies capable of deep penetration and inducing biofilm dispersal. Finally, the chemical composition of plant extracts is highly influenced by factors including source and processing methods, posing significant challenges for standardization and industrialization, particularly regarding batch-to-batch consistency and quality control. The regulatory pathway for plant extract-based veterinary products varies considerably across jurisdictions and depends on the intended use classification, whether as feed additives, veterinary medicinal products, or functional feed ingredients. Understanding and navigating these regulatory requirements represents a critical step in translational development. For products intended for therapeutic claims, such as treatment or prevention of biofilm-associated diseases, registration as veterinary medicinal products typically requires comprehensive demonstration of safety, efficacy, and quality through studies conducted according to regulatory standards. These requirements, while essential for ensuring product reliability and animal safety, also represent substantial investments in time and resources that may pose barriers to development, particularly for products derived from traditional knowledge or intended for limited markets.

The convergence of growing antimicrobial resistance concerns, increasing demand for sustainable animal production, and expanding scientific understanding of plant bioactivity creates a uniquely favorable environment for development of plant extract-based strategies targeting biofilm-associated nutritional and metabolic disorders. Realizing this potential requires sustained, interdisciplinary collaboration among phytochemists, microbiologists, nutritionists, veterinarians, and regulatory scientists, working together to translate fundamental insights into practical solutions that improve animal health, enhance productivity, and support global food security.

## Data Availability

No data was used for the research described in the article.
